# Mitral annular disjunction and its progression during childhood in Marfan syndrome

**DOI:** 10.1093/ehjci/jeae125

**Published:** 2024-05-10

**Authors:** Tam T Doan, Alejandra Iturralde Chavez, Santiago O Valdes, Justin D Weigand, James C Wilkinson, Anitha Parthiban, Sara B Stephens, Ricardo H Pignatelli, Shaine A Morris

**Affiliations:** Division of Cardiology, Texas Children’s Hospital, 6651 Main Street MC-E1920, Houston, TX 77030, USA; Department of Pediatrics, Baylor College of Medicine, One Baylor Plaza, Houston, TX 77030, USA; Division of Cardiology, Texas Children’s Hospital, 6651 Main Street MC-E1920, Houston, TX 77030, USA; Department of Pediatrics, Baylor College of Medicine, One Baylor Plaza, Houston, TX 77030, USA; Division of Cardiology, Texas Children’s Hospital, 6651 Main Street MC-E1920, Houston, TX 77030, USA; Department of Pediatrics, Baylor College of Medicine, One Baylor Plaza, Houston, TX 77030, USA; Division of Cardiology, Texas Children’s Hospital, 6651 Main Street MC-E1920, Houston, TX 77030, USA; Department of Pediatrics, Baylor College of Medicine, One Baylor Plaza, Houston, TX 77030, USA; Division of Cardiology, Texas Children’s Hospital, 6651 Main Street MC-E1920, Houston, TX 77030, USA; Department of Pediatrics, Baylor College of Medicine, One Baylor Plaza, Houston, TX 77030, USA; Division of Cardiology, Texas Children’s Hospital, 6651 Main Street MC-E1920, Houston, TX 77030, USA; Department of Pediatrics, Baylor College of Medicine, One Baylor Plaza, Houston, TX 77030, USA; Division of Cardiology, Texas Children’s Hospital, 6651 Main Street MC-E1920, Houston, TX 77030, USA; Department of Pediatrics, Baylor College of Medicine, One Baylor Plaza, Houston, TX 77030, USA; Division of Cardiology, Texas Children’s Hospital, 6651 Main Street MC-E1920, Houston, TX 77030, USA; Department of Pediatrics, Baylor College of Medicine, One Baylor Plaza, Houston, TX 77030, USA; Division of Cardiology, Texas Children’s Hospital, 6651 Main Street MC-E1920, Houston, TX 77030, USA; Department of Pediatrics, Baylor College of Medicine, One Baylor Plaza, Houston, TX 77030, USA

**Keywords:** mitral annular disjunction, Marfan syndrome, children, aortic root dilation, echocardiography, cardiac magnetic resonance imaging

## Abstract

**Aims:**

Data on mitral annular disjunction (MAD) in children with Marfan syndrome (MFS) are sparse. To investigate the diagnostic yield of MAD by echocardiography and cardiac magnetic resonance imaging (CMR), its prevalence and progression during childhood.

**Methods and results:**

We included patients <21 years old with MFS, defined by 2010 Ghent criteria and a pathogenic *FBN1* variant or ectopia lentis. Two readers measured systolic separation between the mitral valve (MV) posterior hinge point and left ventricular (LV) myocardium on initial and subsequent imaging. MAD was defined as MV-LV separation ≥2 mm, MV prolapse (MVP) as atrial displacement ≥2 mm. Kappa coefficients evaluated echocardiogram–CMR agreement. Bland–Altman and intraclass correlation coefficients (ICCs) assessed inter-rater and inter-modality reliability. Univariable mixed-effects linear regression was used to evaluate longitudinal changes of MAD. MAD was detected in 60% (110/185) eligible patients. MVP was present in 48% (53/110) of MAD and MAD in 90% (53/59) of MVP. MAD detection by CMR and echocardiography had 96% overall agreement (Kappa = 0.89, *P* < 0.001) and a 0.32 mm estimate bias (95% CI 0.00, 0.65). ICC by echocardiography, CMR, and between modalities were 0.97 (95% CI 0.93, 0.98), 0.92 (95% CI 0.79, 0.97), and 0.91 (95% CI 0.85, 0.94), respectively. MAD was associated with aortic root dilation (*P* < 0.001). MAD was found in children of all ages, increased +0.18 mm/year (95% CI +0.14, +0.22) during a median duration of 5.5 years (IQR 3.1, 7.5 years). MAD indexed by height yielded a constant value +0.0002 mm/m/year (95% CI −0.0002, +0.0005 mm/m/year).

**Conclusion:**

MAD was common in pediatric MFS and was associated with aortic root dilation. MAD detection by echocardiography and CMR was highly reliable, suggesting that routine assessment in MFS is feasible. MAD was present in neonates and progressed over time but remained constant when indexing by height. Further studies are needed to evaluate MAD as a biomarker for clinical outcomes in pediatric MFS.

## Graphical Abstract

**Graphical Abstract:**
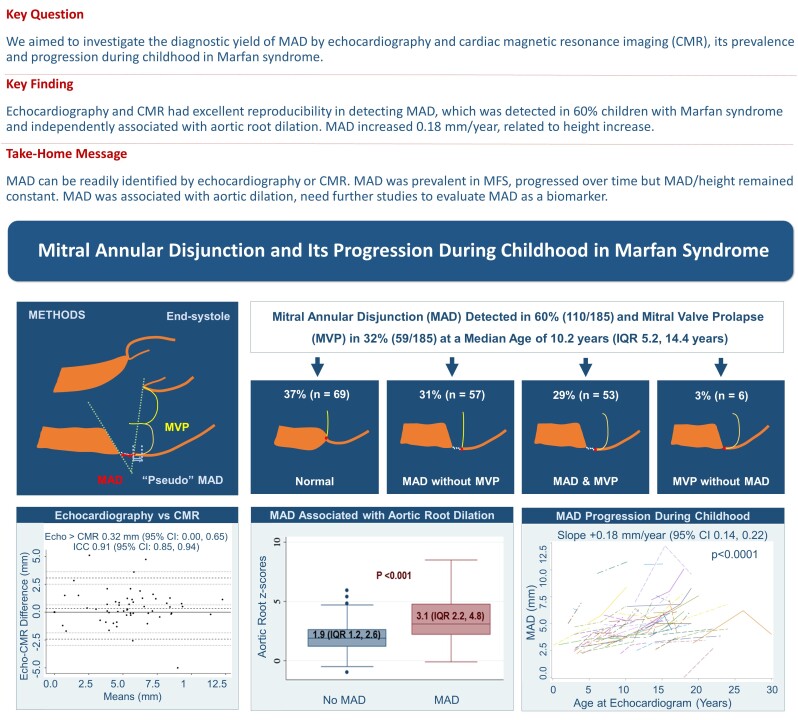

## Introduction

Mitral annular disjunction (MAD) refers to the anatomical separation between the posterior mitral valve (MV) annulus and the crest of the left ventricular (LV) myocardium.^1–4^ This condition is strongly associated with mitral valve prolapse (MVP), a common feature in patients with Marfan syndrome (MFS).^2,5^ The presence of MAD has been associated with higher rates of aortic events, mitral valve surgery, and arrhythmia in patients with MFS.^6–13^

While previous studies have reported MAD to be more prevalent in children than adults with MFS,^8^ it remains unclear whether MAD was an acquired condition and how it progresses during childhood. Additionally, imaging methods used to measure LV–MV separation distance and the criteria used to define MAD are not consistent between studies.^9,13–16^ MAD has been identified using both echocardiography and cardiac magnetic resonance imaging (CMR) in adults, although the agreement on MAD or correlation of LV–MV separation distance between the two modalities has not been widely studied.

In this study, we developed a standardized method to measure LV–MV separation distance and evaluated its reproducibility and MAD agreement between two readers, as well as between echocardiography and CMR. We then investigated the prevalence of MAD, its association with MVP and aortic root dilation, and MAD progression during childhood in pediatric MFS. By doing so, we aimed to establish a foundation for a better understanding of MAD’s impact on children with MFS and further investigations into MAD in other pediatric populations.

## Methods

### Study population

This retrospective study included all patients with MFS under 21 years old seen at Texas Children’s Hospital who had an initial echocardiogram between January 2012 and December 2022. MFS was defined as meeting the 2010 Ghent criteria^17^ as well as either a pathogenic *FBN1* variant or ectopia lentis and no other explanatory genetic conditions. Only patients with an initial echocardiogram prior to mitral valve surgery were included. Patient demographics, including age, sex, race, ethnicity were collected. We excluded patients with suboptimal acoustic windows on their echocardiograms. For a secondary longitudinal analysis, the population was limited to subjects with more than one echocardiogram.

### Echocardiography

Clinically acquired echocardiograms were used for the analysis. Echocardiography was obtained using either a Vivid E9 or E95 system (GE Healthcare) or an IE33 or Epiq CVx ultrasound system (Philips Healthcare) in accordance with institutional protocol and pediatric guidelines.^18^ Echocardiograms available in our institutional Picture Archiving and Communication System were carefully reviewed by A.T.C. and T.T.D.

**Figure 1:**
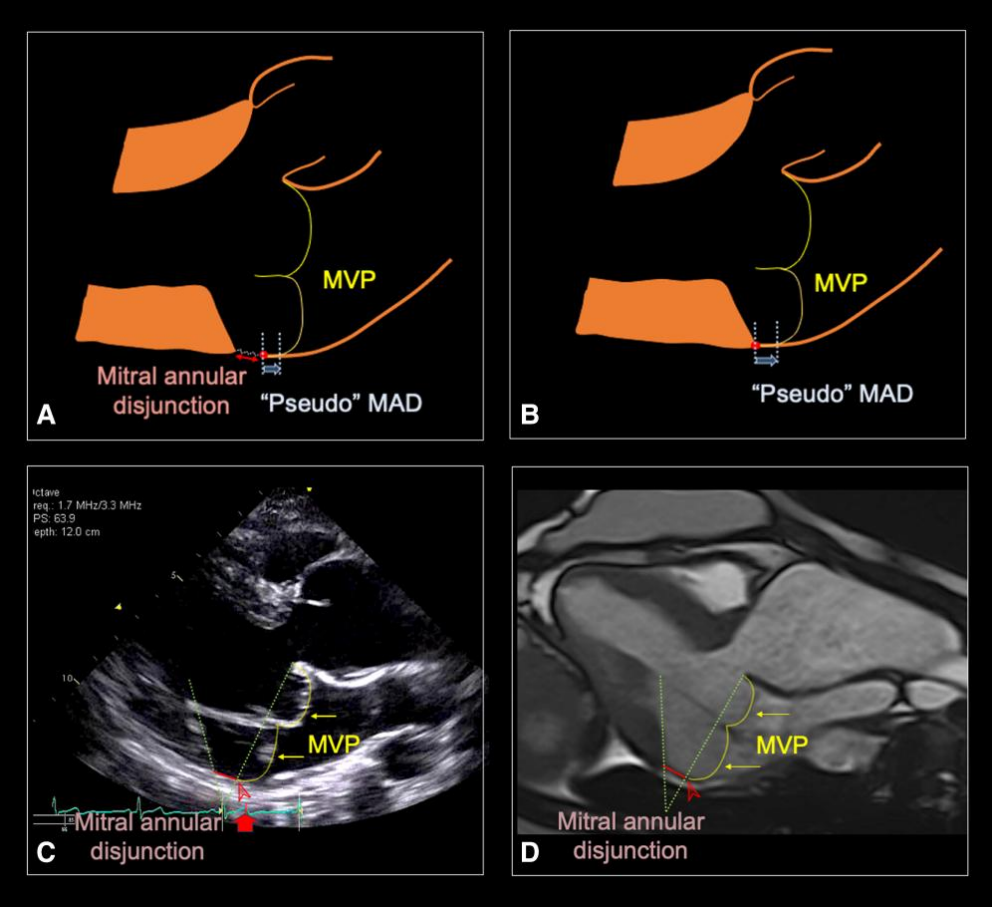
Identifying MAD and ‘pseudo’ MAD in MVP. (*A*) ‘Pseudo’ MAD (arrows) in MVP in a patient with both MVP and MAD (double-ended arrow). (*B*) ‘Pseudo’ MAD in a patient with MVP without MAD. (*C*) MAD in a patient with MVP, measured on echocardiogram at peak T-wave (arrow on the ECG tracing), between the LV myocardium and the posterior MV leaflet hinge point (arrowheads). (*D*) MAD in a patient with MVP, measured on cardiac magnetic resonance imaging. Two dotted lines were drawn, one crossing the two MV leaflet hinge points and a second one along the crest of LV myocardium. The distance between the two lines as they crossed the posterior wall was measured. LV, left ventricular; MAD, mitral annular disjunction; MV, mitral valve; MVP, mitral valve prolapse.

For assessment of the LV–MV separation distance and MVP, we employed the parasternal long axis view to examine the space between the MV posterior leaflet hinge point and the crest of the LV myocardium. In this manuscript, we introduced a standardized method for identifying and measuring the LV–MV separation during systole (see Supplementary data online, *Figure S1*). The analysis involved a meticulous review of the posterior MV hinge point, commencing in the diastole to ensure optimal visualization and tracking of the posterior MV hinge point to avoid overestimating the LV–MV separation distance by including ‘pseudo’ MAD in MVP (*Figure 1A* and *B*).^1,19^ The measurement was made on the imaging frame near or at the peak of the T-waves. To enhance clarity, images were magnified to 200% if deemed necessary to facilitate improved visualization of the region. Two lines were drawn: one connecting the anterior and posterior hinge points of the mitral valve (MV annular plane) and a second line along the crest of the ventricular myocardium, extending across its insertion in the inferolateral wall. Subsequently, the LV–MV separation distance at the inferolateral wall was measured (*Figure 1C*). To account for potential variability among different cardiac cycles, three measurements were taken from three separate cardiac cycles for each patient and then averaged.

The degree of MV regurgitation (none, mild, moderate, or severe), left ventricular ejection fraction (LVEF), aortic root measurements, and *z*-scores was recorded from the initial baseline echocardiographic reports. Low LVEF was defined as ejection fraction <55%. Aortic root dilation was defined as having an aortic root *z*-score > 2.^20,21^ Weight, height, body surface area, and blood pressures were recorded at each echocardiogram.

For longitudinal evaluation, at least two echocardiograms for each patient were selected, including the initial and the last echocardiogram available (before MV intervention). A third echocardiogram between the initial and last echocardiogram, if available, was included in the serial echocardiograms and LV–MV separation distance was measured.

### Cardiac magnetic resonance imaging

We performed a search of the institutional CMR database to include a subset of patients with a CMR examination. All CMR studies were clinically obtained and conducted using a 1.5 T Achieva, 1.5 T Ingenia scanner (Philips Medical System, Best, the Netherlands) or a 1.5 T Aera scanner (Siemens Healthineers, Erlangen, Germany). The initial CMR for each patient with a long axis of the left ventricular outflow tract was examined.

LV–MV separation distance was measured during the end-systolic phase of a cine in the left ventricular outflow tract view. Special attention was given to choose the imaging plane to avoid foreshortening of the LV, minimizing the risk of over or underestimating the LV–MV separation distance. To establish the end-systole, we selected the phase in which the intracavity ventricular blood pool was at its smallest size and the MV remained closed in proximity to aortic valve closure. It was important to track the MV posterior hinge point from diastole into systole. Two lines were drawn: one crossing the two MV hinge points and the second one along the crest of LV myocardium crossing the lateral wall (*Figure 1D*).

For both echocardiography and CMR, we defined MAD as having an LV–MV separation distance ≥2 mm and MVP as atrial displacement of the anterior or posterior leaflet ≥2 mm beyond the MV annular plane at the end-systole. For assessment of inter-modality correlation and agreement, the echocardiogram with the closest date to the CMR was used. A second reader (T.T.D.) independently measured LV–MV separation distance in 40 echocardiograms and 20 CMR studies. Readers were blinded to patients’ clinical data when assessing all echocardiographic and CMR studies.

### Statistical analysis

Categorical variables were presented as number and percentages. The normality of the continuous variables was assessed using Shapiro–Wilk test. Continuous variables were expressed as median and inter-quartile range (IQR) or mean ± standard deviation, where appropriate. χ^2^ and Fisher’s exact test were used to assess for differences between patients with and without MAD.

Patients were stratified into four age quartiles based on the age at their initial echocardiograms. Characteristics of MV abnormalities (MVP, MAD, and MV regurgitation) were described and compared among the groups. Wilcoxon rank sum test and Kruskal–Wallis rank test to compare continuous variables by patients stratified by the 4 age quartiles (0–5.2, 5.2–10.2, 10.2–14.4, and 14.4–21 years old). Chi-square test for trend was used to evaluate differences in frequency of aortic root dilation at initial evaluation by age groups.

To assess the agreement and variability of MAD and LV–MV separation distance measurements between echocardiography and CMR and between the two readers, we utilized Kappa coefficient, Bland–Altman plots, and intraclass correlation (ICC) coefficients. For the 40 echocardiograms and 20 CMR studies independently read by a second reader, inter-rater reliability between two readers was computed using Bland–Altman and ICC analyses. Given potential complex relationships between MAD and MVP and the outcomes of MV regurgitation and aortic root dilation, MAD, MVP, and age (as exposure) were modeled in univariate logistic regressions models and a multivariate logistic regression model (when both were associated) with these outcomes on first echocardiogram.

To analyze the longitudinal trends in LV–MV separation distance during childhood among patients with more than one echocardiogram, we then conducted univariable mixed-effects linear regression using age as the exposure, LV–MV separation as the outcome, and patient ID as a random effect to account for repeated measures. Recognizing the often non-linear associations of imaging parameters with BSA in children, we adjusted MAD to BSA to the power of 0.5 as consistent with linear measurements of cardiac structures prior literature.^22^ Separate spaghetti plots of age, height, and BSA (x-axis) vs. crude MAD (y-axis) were generated for visualization. Mixed-effects linear regression was then repeated, changing the exposure to BSA, age and then height and best fits were compared between models when evaluating crude MAD. Coefficients delineating annual change in MAD generated from linear regression models were compared between patients with and without MVP. Conversely, coefficients generated on regression models of BSA-adjusted, age-adjusted, and height-adjusted MAD were separately compared by MVP diagnosis. A two-sided *P*-value <0.05 was considered statistically significant. All analyses were performed using SAS (version 9.2 copyright 2002–2008, SAS, Cary, NC).

## Results

### MAD by echocardiography

**Figure 2:**
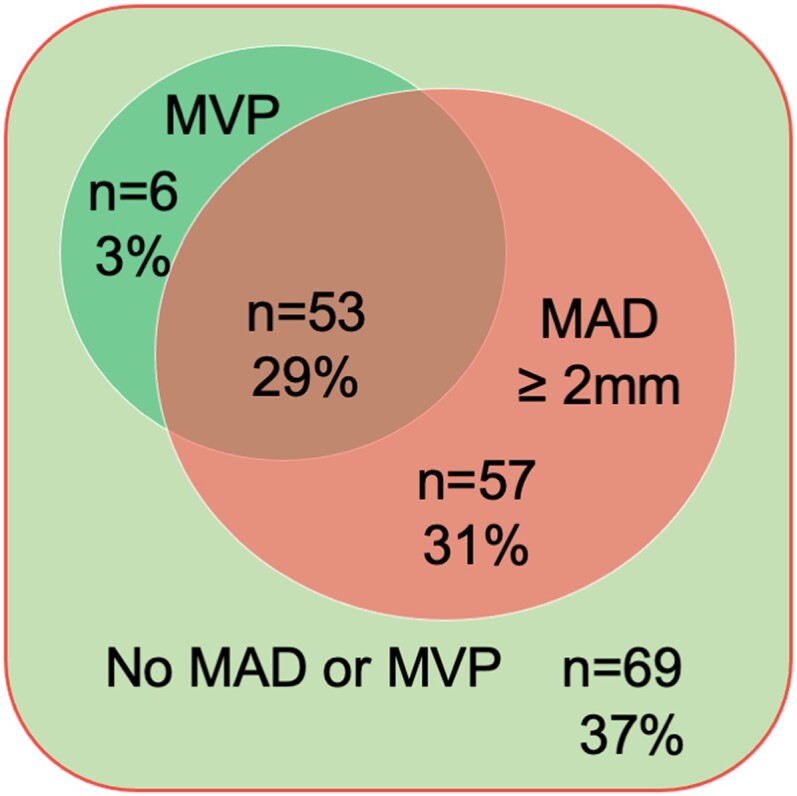
Venn diagram of mitral valve prolapse (MVP) and mitral annular disjunction (MAD) in 185 patients <21 years old with Marfan syndrome.

After excluding three subjects whose first available echocardiograms were after their MV surgery, we included 185 pediatric patients with MFS (54% male) with initial echocardiogram at a median age of 10.2 years (IQR 5.2, 14.4 years) (*Table 1*). One-hundred sixty-four patients (89%) received an MFS diagnosis via a documented pathogenic *FBN1* variant. Eighty (44%) had ectopia lentis (including 59 patients also with pathogenic *FBN1* variant). MAD was detected on the initial echocardiogram in 60% (110/185) of patients, while MVP was less common and present in nearly one-third of patients (32%, 59/185; *Figure 2*). MAD was detected at a similar rate in all four age quartiles, ranging from 51 to 66%. Among those with MAD, MVP was found in 48% (53/110) whereas MAD was present in 90% (53/59) of those with MVP. LVEF was significantly lower among patients with MAD compared with those without. All five patients (3%) with low LVEF had MAD (*Table 1*). Aortic root dilation was present in 64% (118/185) patients and moderate-to-severe MV regurgitation was present in 14% (25/185) of patients on their first echocardiograms.

**Table 1:** Baseline characteristics of patients with and without mitral annular disjunction on their initial echocardiograms

Variable	Total	MAD	No MAD	*P*-value
	*n* = 185	*n* = 110	*n* = 75	
Male, *n* (%)	99 (54)	54 (49)	45 (60)	0.144
Age in years (IQR)	10.2 (5.2, 14.4)	10.1 (5.2, 14.0)	10.3 (5.1, 14.8)	0.739
Race–ethnicity, *n* (%)				0.159
Non-Hispanic White	85 (46)	52 (47)	33 (44)	
Non-Hispanic Black	18 (10)	15 (14)	3 (4)	
Hispanic	60 (32)	32 (29)	28 (37)	
Asian	11 (6)	6 (5)	5 (7)	
Not reported	11 (6)	5 (5)	6 (8)	
Weight in kg (IQR)	39.5 (20.5, 65.8)	34.3 (20.1, 63.5)	49.5 (22.2, 70.0)	0.101
Height in cm (IQR)	158 (123, 178)	155 (127, 176)	154 (120, 177)	0.499
BSA in m^2^ (IQR)	1.34 (0.83, 1.80)	1.20 (0.86, 1.71)	1.17 (0.81, 1.78)	0.171
Blood pressure				
Systolic in mmHg ± SD	106 ± 14	105 ± 14	107 ± 14	0.591
Diastolic in mmHg ± SD	62 ± 9	61 ± 9	63 ± 8	0.221
Documented *FBN1* variant, *n* (%)	164 (89)	96 (87)	68 (91)	0.991
Ectopia lentis, *n* (%)	80 (44)	45 (42)	35 (49)	0.358
MVP, *n* (%)	59 (32)	53 (48)	6 (8)	<0.001
Bi-leaflet	42 (23)	41 (37)	1 (1)	<0.001
Anterior leaflet only	7 (4)	3 (3)	4 (5)	0.444
Posterior leaflet only	10 (5)	9 (8)	1 (1)	0.051
MV regurgitation severity, *n* (%)				<0.001
None–mild	160 (86)	87 (79)	73 (97)	
Moderate–severe	25 (14)	23 (21)	2 (3)	
LVEF in % (IQR)	63 (60, 66)	62 (59, 65)	64 (60, 67)	0.024
LVEF < 55%	5 (3)	5 (5%)	0	0.082
Aortic root in mm (IQR)	30.6 (24.7, 35.1)	31.2 (26.6, 36.2)	28.0 (23.3, 34.3)	0.014
Aortic root *z*-score (IQR)	2.5 (1.6, 3.9)	3.1 (2.2, 4.8)	1.9 (1.2, 2.6)	<0.001
*z*-score ≥ 2, *n* (%)	118 (64)	84 (76)	34 (45)	<0.001
Age Quartile 1 (<5.2 years)	3.1 (2.2, 4.8)	3.7 (2.9, 5.5)	2.9 (2.0, 3.5)	0.043
Age Quartile 2 (5.2–10.2 years)	2.5 (1.9, 3.9)	3.1 (2.2, 4.3)	2.2 (1.2, 2.5)	0.006
Age Quartile 3 (10.2–14.4 years)	2.4 (1.3, 3.8)	3.3 (2.2, 4.9)	1.4 (0.7, 2.0)	<0.001
Age Quartile 4 (14.4–21.0 years)	2.0 (1.1, 2.8)	2.6 (1.3, 4.3)	1.8 (1.0, 2.2)	0.013

BSA, body surface area; IQR, inter-quartile range; LVEF, left ventricular ejection fraction; MAD, mitral annular disjunction; MV, mitral valve; MVP, mitral valve prolapse.

On univariable analyses, both MAD and MVP were associated with moderate–severe MV regurgitation and aortic root dilation (*Tables 1* and *2*). However, multivariable analysis showed only MVP was independently associated with moderate–severe MV regurgitation (OR 11.73, 95% CI 3.53, 38.96, *Table 2*). Conversely, only MAD (OR 3.64, 95% CI 1.75, 7.56) and age (OR 0.89, 95% CI 0.84, 0.95) were independently associated with aortic root dilation.

**Table 2:** Association between MAD, MVP, and age with MV regurgitation, LVEF, and aortic root dilation on initial echocardiograms

Outcomes	MAD OR (95% CI)	*P*	MVP OR (95% CI)	*P*	Age OR* (95% CI)	*P*
Univariable analysis						
MV regurgitation	9.65 (2.20, 42.30)	0.003	16.85 (5.44, 52.15)	<0.001	0.98 (0.91, 1.05)	0.606
Aortic root dilation (*z*-score > 2)	3.89 (2.07, 7.33)	<0.001	2.06 (1.04, 4.09)	0.038	0.89 (0.86, 0.95)	<0.001
Multivariable analysis						
MV regurgitation	3.25 (0.65, 16.20)	0.150	11.73 (3.53, 38.96)	<0.001	0.96 (0.88, 1.06)	0.498
Aortic root dilation (*z*-score > 2)	3.64 (1.75, 7.56)	0.001	1.23 (0.54, 2.76)	0.614	0.89 (0.84, 0.95)	0.001

LVEF, left ventricular ejection fraction; MV, mitral valve; CI, confidence interval; MAD, mitral annular disjunction; MVP, mitral valve prolapse.

* ORs expressed per one unit increase.

**Figure 3:**
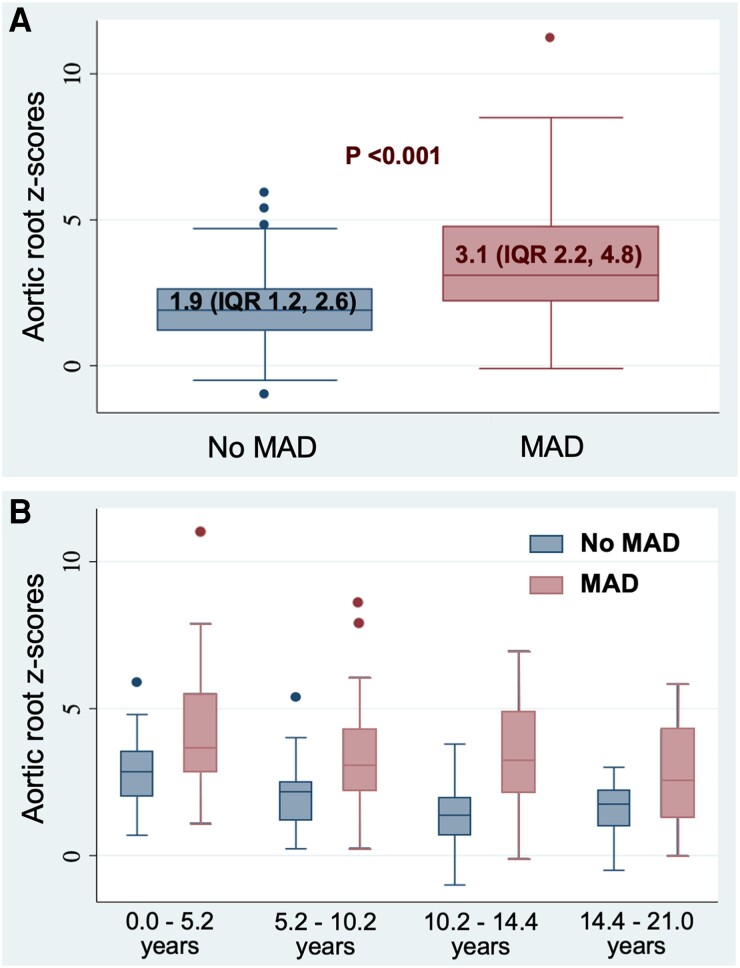
Aortic root *z*-scores between Marfan syndrome patients with and without mitral annular disjunction (*A*) and among the four age quartiles (*B*). MAD, mitral annular disjunction.

Aortic root *z*-scores at first echocardiogram were the highest in the youngest groups and decreasing with advancing age (*Table 3*), suggesting those with the largest aortas presented at the youngest ages. Moreover, larger aortic root *z*-scores were noted in patients with MAD in the entire cohort and in each of the four age quartiles (*Table 1*, *Figure 3*).

**Table 3:** Mitral valve abnormalities and aortic root *z*-scores on the first echocardiogram by age quartiles

Characteristics	IQR1 (*n* = 46)	IQR2 (*n* = 45)	IQR3 (*n* = 47)	IQR4 (*n* = 47)	*P*-value
	<5.2 years	5.2 to <10.2	10.2 to <14.4	14.4 to <21	
Male, *n* (%)	28 (61)	18 (40)	24 (51)	29 (62)	0.128
MAD, *n* (%)	27 (59)	28 (62)	31 (66)	24 (51)	0.503
LV–MV distance, mm (IQR)	2.3 (0.0, 3.7)	3 (1.3, 4.9)	3.9 (0.0, 5.1)	3.0 (0.0, 5.0)	0.231
MVP, *n* (%)	9 (20)	21 (47)	18 (38)	11 (23)	0.017
Bi-leaflet	7 (15)	14 (31)	15 (32)	6 (13)	0.042
Posterior leaflet only	0	6 (13)	2 (4)	2 (4)	0.035
Anterior leaflet only	2 (4)	1 (2)	1 (2)	3 (6)	0.783
MV regurgitation, *n* (%)					0.023
None to mild	42 (91)	34 (76)	39 (83)	45 (96)	
Moderate to severe	4 (9)	11 (24)	8 (17)	2 (2)	
Aortic root *z*-score, IQR	3.1 (2.2, 4.8)	2.5 (1.9, 3.9)	2.4 (1.3, 3.8)	2.0 (1.1, 2.8)	0.002
*z*-score ≥ 2, *n* (%)	36 (78)	32 (71)	28 (60)	22 (47)	0.0008

IQR, inter-quartile range; LV–MV, left ventricle to mitral valve separation; MAD, mitral annular disjunction; MV, mitral valve; MVP, mitral valve prolapse.

### Assessment of agreement and reliability

**Figure 4:**
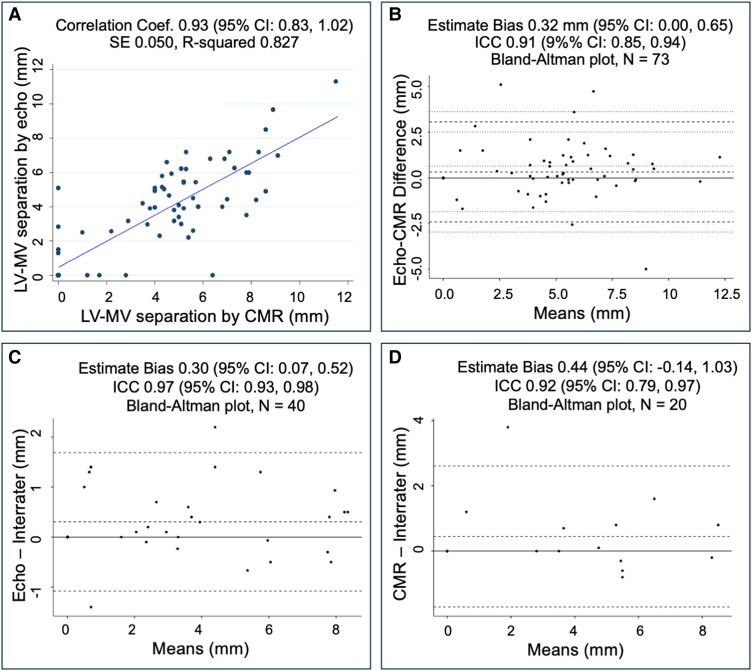
The agreement between echocardiography (echo) and cardiac magnetic resonance (CMR) imaging on the separation distance between the left ventricular (LV) crest and mitral valve (MV) posterior hinge point (*A*) Pearson correlation and (*B*) Bland–Altman plot and inter-rater variability by echocardiography (*C*) and by CMR (*D*). ICC, intraclass correlation. 95% CI, 95% confidence interval.

A total of 73/185 (40%) patients, including 53/73 (73%) with MAD by echocardiography, had both echocardiography and CMR done within 6 months (IQR 2–14 months). Echocardiography and CMR yielded an overall percent agreement of 96% (Kappa = 0.89, *P* < 0.001) in detecting MAD. When considering CMR as the gold standard to diagnose MAD, echocardiography detected MAD with a sensitivity of 100% (95% CI 100%, 100%) and specificity of 86% (95% CI 77.5%, 93.9%). Echocardiography detected MAD in three patients in whom MAD was not apparent on CMR. The LV–MV separation distance had an inter-modality correlation coefficient of 0.93 (95% CI 0.83, 1.02), indicating high degree of agreement between the two modalities (*Figure 4A*). Additionally, LV–MV separation distance had an estimate bias of 0.32 mm (95% CI 0.00, 0.65 mm; *Figure 4B*), with measurements by echocardiography slightly greater than those obtained by CMR. The ICC of LV–MV separation distance between two raters was 0.97 (95% CI 0.93, 0.98) by echocardiography and 0.92 (95% CI 0.79, 0.97) by CMR (*Figure 4C* and *D*).

### MAD progression over time

A total of 98/185 (53%) patients, including 63/110 (57%) with MAD had serial echocardiograms available for longitudinal evaluation over a median follow-up of 5.3 years (IQR 3.4, 7.2 years). Aortic root *z*-scores on the initial echocardiogram between patients with and without serial echocardiograms were not different (*P* = 0.823). Their last echocardiogram was performed at a median age of 14.9 years (IQR 9.8, 18.6 years). The two youngest patients with MAD were neonates (<28 days), and an additional eight patients had MAD detected within the first year of life, which accounted for 9% (10/110) of all MAD cases. MAD was present on subsequent echocardiogram in all patients who had MAD on their first echocardiogram. None of the patients without MAD on their initial echocardiogram developed it on subsequent echocardiograms. In the 63 patients with MAD and follow-up echocardiograms, MVP was present in 56% (35/63).

During a median of 5.5 years (IQR 3.1, 7.5 years), MAD increased on average +0.18 mm/year (95% CI +0.14, +0.22 mm/year; Supplementary data online, *Figure S2A*). This increase was faster among patients with MVP, with an average increase of +0.21 mm/year (95% CI +0.15, +0.26 mm/year) among those with MVP compared with +0.10 mm/year (95% CI +0.04, +0.15 mm/year) among those without MVP. A significant increase in MAD was also noted with increasing BSA and increasing height (see Supplementary data online, *Figure S2B* and *C*). Given these association, adjustment of MAD by these indices was explored to determine if there were any stable associations. MAD adjusted by age significantly decreased over time (−0.11 mm/year, 95% CI −0.17, −0.05 mm/year). When evaluating changes in BSA-adjusted MAD, a downslope of −0.036 mm/year (95% CI −0.0008, −0.073 mm/year) was observed, which bordered on statistical significance (*P* = 0.055). MAD adjusted by height remained constant over time (annual change of +0.0002 mm/m, 95% CI −0.0002, +0.0005 mm/m annual change; *Table 4*), as well as stayed constant in those with MVP, suggesting that MAD indexed for height is a stable indexing measure over time.

**Table 4:** Progression of mitral annular disjunction during childhood in Marfan syndrome

MAD groups	MAD Dimension			BSA^0.5^-adjusted MAD^a^		
	Slope mm/year	95% CI mm/year	*P*-value	Slope mm/BSA^0.5^/year	95% CI mm/BSA^0.5^/year	*P*-value
All patients (*n* = 63)	0.18	0.14, 0.22	<0.0001	0.036	−0.00075, 0.073	0.055
MVP (*n* = 35)	0.21	0.15, 0.26	<0.0001	0.061	0.0095, 0.11	0.021
No MVP (*n* = 28)	0.10	0.04, 0.15	0.0018	−0.017	−0.072, 0.038	0.538

MAD groups	Age-adjusted MAD			Height-adjusted MAD		
	Slope mm/year^2^	95% CI mm/year^2^	*P*-value	Slope mm/m/year	95% CI mm/m/year	*P*-value
All patients (*n* = 63)	−0.11	−0.17, −0.05	0.0002	0.0002	−0.0002, 0.0005	0.317
MVP (*n* = 35)	−0.16	−0.26, −0.06	0.0017	0.0003	−0.0001, 0.001	0.120
No MVP (*n* = 28)	−0.06	−0.09, −0.04	<0.0001	−0.0002	−0.0006, 0.0003	0.459

BSA, body surface area; CI, confidence interval; MAD, mitral annular disjunction; MVP, mitral valve prolapse.

^a^BSA^0.5^: adjusted MAD was computed using the following formula: MAD divided by Haycock BSA to the power of 0.5. The *P*-value of the slope tests if the slope is significantly different than 0.

## Discussion

To investigators’ knowledge, this is the first study reporting MAD and MV characteristics in a large population of pediatric patients with MFS. We strongly believe that the introduction of a standardized and reproducible method to identify, measure, and monitor MAD progression on echocardiography and CMR has significant implications in clinical practice and research in pediatric patients with MFS as well as lays a foundation to study MAD in other pediatric populations. The study demonstrated that MAD was identified by transthoracic echocardiography with a high sensitivity and specificity compared with CMR. The two modalities agreed well on the presence or absence of MAD. Our findings support that transthoracic echocardiography is non-inferior to CMR in the detection of MAD, with superior inter-rater reproducibility in pediatric patients. This may be secondary to the superior temporal resolution of echocardiography compared with CMR and its excellent spatial resolution in children.

Findings revealed a higher prevalence of MAD (60%) in the pediatric population compared with reports in adult MFS population (26% to 46%).^6,8^ Remarkably, MAD was detected even in neonates, challenging the notion of an acquired condition and suggesting a higher prevalence in more severe forms of MFS that typically present in younger patients.^6,8^ It was interesting that the high prevalence rate of MAD (90%) in pediatric MFS patients with MVP was similar to Hutchins’ observation of cardiac specimens in deceased older adults (92%, 22/25).^2^ As a result, MVP was independent of MAD in only a few cases (*n* = 6 or 10% of MVP), reiterating the importance of careful evaluation of MAD in individuals with MVP for an accurate characterization.^19^ It remains to study the implication of these rare entities and if pediatric patients who had MVP without MAD would develop MAD in adulthood.

Progressive aortic root dilation and aortic dissection are the leading cause of death in MFS.^23^ Our results showed higher aortic root *z*-scores in patients with MAD compared with those without MAD in the entire study population and in all four age quartiles. This observation was similar to a study in adults with MFS that aortic root mean *z*-score was 3.5 in patients with MAD vs. 2.0 in patients without MAD.^8^ In addition, our study revealed aortic root dilation was not associated with MVP or patients’ age but was independently associated with MAD, suggesting that MAD may be an important biomarker in the management of young patients with MFS with respect to aortic root dilation. More patients in the first and second age quartiles (0 to 10.2 years) had aortic root dilation compared with older children on their first echocardiogram, likely demonstrating a more severe phenotype in younger patients with pediatric MFS. It would be important to study aortic root size longitudinally to better understand how MAD affects its natural history.

Primary cardiomyopathy has been recognized as a separate entity that may be present in patients with MFS, independent of left heart volume overload from MV regurgitation, reported more commonly in adults than pediatric patients.^24^ In our study, a trend of lower LVEF in patients with MAD raised a question if MAD was associated with substrates of cardiomyopathy reported patients with MFS. Exploring the association of MAD with markers of cardiomyopathy in larger population of pediatric patients with MFS would provide additional insights to the phenotypical profile of MFS.

Additionally, this study delineates the natural history of MAD in pediatric patients with MFS. In particular, 9% of MAD cases (10/110) were detected among neonates and infants, suggesting that MAD is possibly a congenital abnormality. In pediatric patients with MAD, its absolute distance increased during childhood, slightly more so in patients with MVP compared with those without MVP. These results diverge from prior observations by Demolder and colleagues that unadjusted MAD distance had no significant changes over time.^8^ This may have been related to the underrepresentation of pediatric patients in their study compared with our study population, and given the strong association with height, the MAD distance may only increase with somatic growth then stop as cardiac growth stops. We observed that MAD did not develop from an initially normal appearing LV–MV junction in a subset of patients with available subsequent imaging during childhood. It remains unclear if MAD would develop in adults with MFS who did not have MAD during childhood and how MAD affects the natural history of patients with MFS. It would be important to study aortic root growth rate longitudinally to better understand how MAD affects its natural history.

### Limitations

This study was limited by retrospective design at a single tertiary center, presenting possible referral bias. Serial echocardiographic studies were only available in 53% of all patients. Although one may have thought more echocardiograms were available in patients with more severe disease, we have observed no difference in aortic root *z*-scores on the initial studies between those who had serial echocardiograms and those who did not. There is a lack of individual follow-up echocardiography and CMR both in younger patients and infants with more severe phenotypes of MFS and in patients without significant MV regurgitation or aortic root dilation.

## Conclusions

Both echocardiography and CMR were reproducible modalities to detect MAD with echocardiography being comparable to CMR, likely owing to its excellent temporal and spatial resolutions in children. MAD was highly prevalent in pediatric patients with MFS at similar rates throughout the four age quartiles. MVP was present in nearly one-half of patients with MAD whereas MAD was present in 90% of patients with MVP. MAD was associated with aortic root dilation and lower LVEF. MAD distance increased during childhood, more so in patients with MVP, and the increase was linearly related to increase in individual heights, and the relationship relative to the height was stable over time. MAD should be included in the routine assessment of pediatric patients with MFS although additional investigations including longitudinal studies are necessary to better understand the roles of MAD as a biomarker in disease progression patterns of pediatric patients with MFS. Future research endeavors should explore whether indexed MAD could serve as a novel biomarker for clinical outcomes in pediatric MFS.

## Supplementary data

Supplementary data are available at *European Heart Journal - Cardiovascular Imaging* online.

## Supplementary Material

jeae125_Supplementary_Data

## Data Availability

The data underlying this article will be shared on reasonable request to the corresponding author.
