# Supplementary material for: Mitral annular disjunction and its progression during childhood in Marfan syndrome
Source: Eur Heart J Cardiovasc Imaging. 2024 May 10;25(9):1306–14. doi: 10.1093/ehjci/jeae125 (PMC11346360; doi:10.1093/ehjci/jeae125)
Supplement: jeae125_Supplementary_Data [file jeae125_supplementary_data.docx]

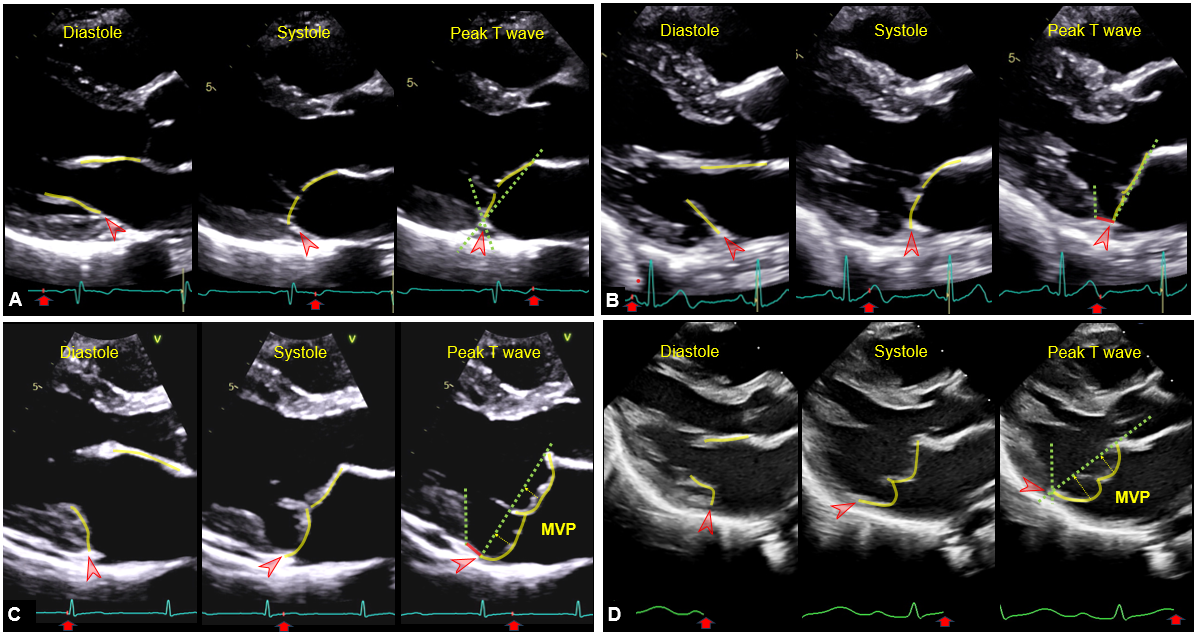


Supplemental Figure 1. Identification and classification of mitral annular disjunction (MAD) and mitral valve prolapse (MVP) in Marfan syndrome. The MV posterior hinge point (red arrow heads) was followed starting in diastole into systole near peak T waves (red arrows). (A) no MAD and no MVP. (B) MAD without MVP. (C) MAD and MVP. (D) MVP without MAD.


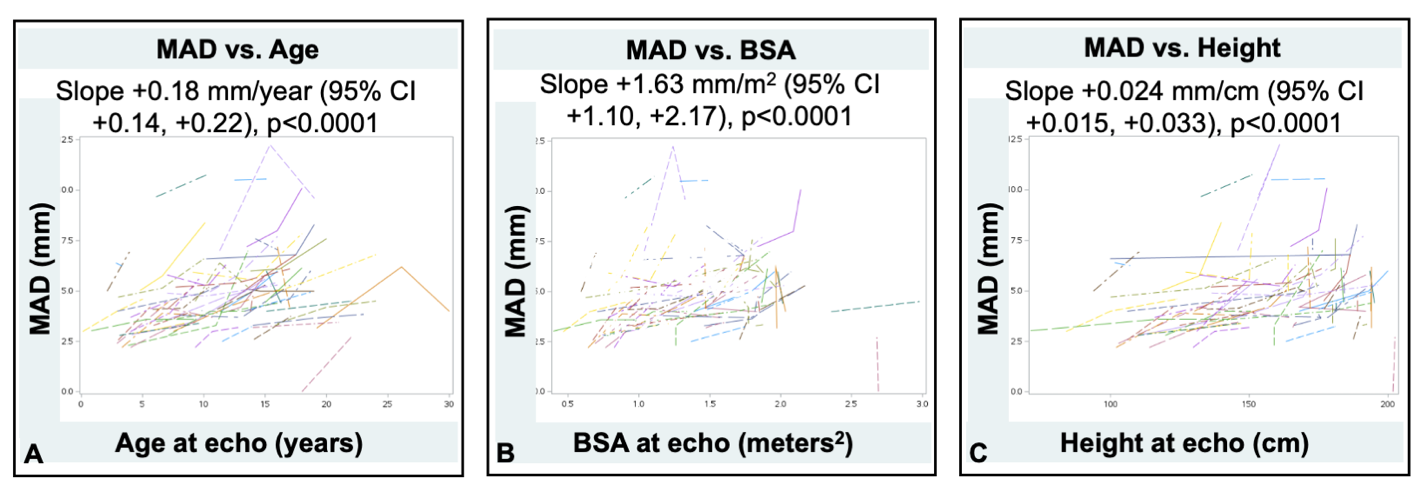


Supplemental Figure 2. Spaghetti Plots of Longitudinal Trends in MAD distance (A) and MAD distance indexing by BSA^0.5^ (B) and height (C).
